# Status of HIV-infected patients classified as lost to follow up from a large antiretroviral program in southwest Nigeria

**DOI:** 10.1371/journal.pone.0219903

**Published:** 2019-07-25

**Authors:** Mobolanle Balogun, Seema Thakore Meloni, Ugonnaya Ugochinyere Igwilo, Alero Roberts, Ifeoma Okafor, Adekemi Sekoni, Folasade Ogunsola, Phyllis J. Kanki, Sulaimon Akanmu

**Affiliations:** 1 Department of Community Health and Primary Care, College of Medicine of the University of Lagos, Lagos, Nigeria; 2 Department of Immunology and Infectious Diseases, Harvard T.H. Chan School of Public Health, Boston, Massachusetts, United States; 3 Department of Community Health, Lagos University Teaching Hospital, Lagos, Nigeria; 4 Department of Medical Microbiology and Parasitology, College of Medicine of the University of Lagos, Lagos, Nigeria; 5 Department of Haematology and Blood Transfusion, College of Medicine of the University of Lagos, Lagos, Nigeria; NPMS-HHC CIC / LSH&TM, UNITED KINGDOM

## Abstract

**Background:**

Loss to follow-up (LTFU) is a term used to classify patients no longer being seen in a clinical care program, including HIV treatment programs. It is unclear if these patients have transferred their care services elsewhere, died, or if there are other reasons for their LTFU. To better understand the status of patients meeting the criteria of LTFU, we traced a sample of HIV-infected patients that were LTFU from the Lagos University Teaching Hospital (LUTH) antiretroviral program.

**Methods:**

We conducted a cross-sectional study of HIV-infected adult patients who enrolled for care between 2010 and 2014 at LUTH and were considered LTFU. Patients with locator information were traced using phone calls. Face-to-face interviews were used to collect data from successfully traced and consenting participants. Predictors of LTFU from LUTH, disengagement from care and willingness to re-engage in care in LUTH were assessed.

**Results:**

Of 6108 registered patients, 3397 (56%) were LTFU and being unmarried was a predictor of being LTFU from LUTH. Of 425 patients that were traced, 355 (84%) were alive and 70 (16%) were dead. Two hundred and sixty-eight patients consented to interviews; 96 (35.8%) of these had transferred to another clinic for care while 172 (64.2%) were disengaged from care. More than half (149/268; 55.6%) were not on antiretroviral therapy (ART). Some of the primary reasons for LTFU were; long distance to clinic (56%) and feeling healthy (6.7%). Predictor of disengagement from care within the interviewed cohort was not having started ART. The predictors of willingness to re-engage in care included, not having started ART, male sex and longer duration in HIV care prior to LTFU.

**Conclusion:**

Most of the interviewed cohort that was LTFU were truly disengaged from care and not on ART. Interventions are required to address processes of re-engagement of patients that are LTFU.

## Introduction

Of the estimated 36.7 million people living with human immunodeficiency virus (HIV), about 20.9 million were on antiretroviral therapy (ART) by the middle of 2017 [1]. Nigeria’s HIV epidemic is the second largest globally, while its new HIV infection rate is among the highest in Africa’s sub-Saharan region [2]. By 2016, about 3.2 million people were living with HIV in Nigeria with the highest prevalence in the southern states of the country [2]. It is estimated that only 31% of the adults and 21% of the children living with HIV are on ART [2].

In recognition that the nation was one of the hardest hit countries both globally and in the African region, the Nigerian government implemented one of the region’s largest ART programs and subsequently received support from the United States President’s Emergency Plan for AIDS Relief (PEPFAR) and the Global Fund, leading to free, HIV care services, laboratory investigations, and ART drugs [3,4]. There are now about 200 facilities providing comprehensive ART services across the nation [5].

With the growth of HIV programs, there is an increased focus on measures to sustain long-term ART benefits and reduce new HIV infection rates [5]. Sustained retention of patients in ART programs is essential for combating the HIV epidemic and for the success of the programs [6,7]. However, the retention in care among patients enrolled in ART programs in sub-Saharan Africa is generally poor, about 60% by the end of the second year, as noted by Rosen et al in their systematic review [7].

Various outcomes have been described for patients who were classified as lost to follow-up (LTFU) from ART programs: death, withdrawal from care, or transfer to other facilities [7–9]. Patient tracing has helped to further characterize the true status of patients classified as LTFU [9], sometimes with the positive effect of reengagement in care [10]. Although patient tracing has been explored widely in Africa [9], few studies in Nigeria have been documented despite the heavy burden of HIV in the country and the well-documented high rates of LTFU [3,11–13]. Questions still remain concerning the magnitude of LTFU in HIV programs and the contributory factors in the densely populated and industrialized region of southwest Nigeria.

In the AIDS Prevention Initiative in Nigeria (APIN), Public Health Initiatives (PHI) and PEPFAR HIV program at the Lagos University Teaching Hospital (LUTH), over 15,000 patients have been enrolled into care since October 2004. Of these, only 8,000 patients remain actively in care. In this study, we used routine clinic data to describe LTFU and identify patients at high-risk. To better characterize reasons for LTFU in the LUTH patient setting, we investigated a sample of HIV-infected patients that were lost to follow-up from the program and determined their current status.

## Methods

### Study site, design and population

The study was carried out in Lagos, southwest Nigeria, between January and November 2017. We conducted a cross-sectional study on patients that had been identified as LTFU using electronic medical record data from the HIV treatment program at LUTH.

This program was officially established with the help of a Harvard-PEPFAR grant (U51HA025522) in October 2004. Patients are enrolled into care following confirmation of HIV infection. The total enrollment into HIV care services at LUTH has grown from 759 patients in 2004 to over 20,000 patients as at the end of December 2018. The clinic has a dedicated laboratory that offers viral load and CD4+ cell count (for free) as well as hematology and chemistry assays (at a cost to patients) that are done at baseline and every 6 months thereafter. The majority of patients receive their HIV prescriptions monthly, while those with stable undetectable viral loads are switched to receiving 3 month prescriptions. Prior to 2016, when the test and treat strategy commenced [14], patients not yet on ART were seen in the clinic every 6 months.

The study population was laboratory-confirmed HIV-infected adult patients who enrolled into care between January 1, 2010 and December 31, 2014 and were LTFU from the clinic. The inclusion criterion for tracing was complete locator information (11-digit phone number) in the electronic medical records system [15]. Patients who were known to have died or known to have been transferred out while still in active care were excluded from the study. During the tracing process, patients who had moved out of state were also excluded from the study due to logistical reasons.

### Definition of variables

We defined LTFU as patients who were lost to care that did not receive any clinical, laboratory or pharmacy services for at least 6 months and did not later return to the clinic by December 31, 2016. Our consideration for this definition was to minimize the false-positive rate (i.e., the proportion of individuals who were considered LTFU who later returned to care) [16]. Retention in care was defined as receiving continued HIV care at any HIV clinic while disengagement from care was defined as not receiving continued HIV care services at an HIV clinic [6].

### Data collection

Three research assistants with postgraduate public health degrees were recruited and trained on the study implementation. As a training and tool refinement opportunity, we conducted a pilot study on a cohort of 25 patients that were LTFU after which we refined the tracing process and the data collection tool; data from the pilot study cohort are not included in this evaluation. Eligible LTFU patients were first contacted by phone to explain the study and arrange appointments for face-to-face interviews. Tracing was deemed successful if the patient could be reached by phone or if an informant familiar with patient could be reached to confirm if the patient was alive or dead. At least three phone call attempts were made on different days and at different times of the day without success before tracing was considered unsuccessful.

Patient tracing and data collection was conducted between June and November 2017. Following collection of written informed consent, research assistants used a structured questionnaire [17–19] (questionnaire in S1 Appendix) in face-to-face interviews to collect information on: socio-demographic characteristics (age, sex, marital status, religion, ethnicity, education and occupation); reasons for discontinuation of care; and, use of antiretroviral medication, including if they were placed on ART at the clinic, if they were still on ART, source of medication, reasons for non-use of ART and adherence to ART. Patients were also asked about registration for care at another clinic and their willingness to return to care in LUTH.

The reasons for discontinuation in care were divided into 5 main categories: access to care; clinic quality; work and family; medical; and alternative treatment and advice [16]. In addition, patients ranked their top 3 reasons for discontinuation.

### Data analysis

Data were analyzed using Stata version 15.1 (StataCorp, USA). Continuous variables were tested for the assumption of normality. Categorical variables were presented in frequencies and percentages while non-normal continuous variables were presented as median and interquartile range (IQR). For the clinic population, Chi-square and Wilcoxon rank sum tests were used to determine differences in patient characteristics within loss to follow-up groups (LTFU vs. not LTFU, traced vs. not traced, traced and interviewed vs. traced and not interviewed). Time to LTFU was modelled using survival analysis methods. All patients that remained in care were right censored. Univariable and multivariable Cox proportional hazards (PH) regressions were conducted to examine associations between baseline patient characteristics and LTFU. Backward elimination was used to derive a parsimonious model. However, some variables such as age and enrollment year were chosen *a priori*. Adjusted and unadjusted hazard ratios (HRs) and 95% confidence intervals (CIs) were computed for each predictor variable. Schoenfeld’s global test of residuals was used to assess for violation of the PH assumption; variables that violated the PH assumption were excluded from the final model.

For the LTFU patients that were successfully traced and interviewed, we used Chi-square and Wilcoxon rank sum tests to evaluate bivariate associations between independent variables (socio-demographic characteristics, months in care prior to LTFU and ART initiation in LUTH) and dependent variables (disengagement from care and willingness to re-engage in the LUTH ART program). Univariable and multivariable binary logistic regression were conducted to examine associations between respondents’ characteristics and disengagement from care and willingness to re-engage in the ART program from which they were LTFU. All independent variables in bivariate analyses were considered for inclusion in the logistic regression analyses irrespective of statistical significance. We used the Box-Tidwell test to test the assumption of linearity between continuous predictor variables (age and months in care prior to LTFU) and the dependent variables, which generated power transformed predictor variables that were included in the logistic regression model. Crude and adjusted odds ratios (OR) and 95% CI were computed for each predictor variable. The Hosmer-Lemeshow’s goodness of fit test was performed. Two-tailed test of hypothesis was assumed. Level of significance was set at 0.05.

### Ethics

The study protocol was approved by the Health Research Ethics Committee (HREC) of the College of Medicine of the University of Lagos (CM/HREC/03/16/006) and the Harvard T. H. Chan School of Public Health Institutional Review Board. This study only included patients that provided voluntary consent for use of their clinic data for future research purposes. Written informed consent was collected from traced patients prior to interviews and those that indicated willingness to return to LUTH were linked back to care.

## Results

### Outcome of tracing

**Fig 1:**
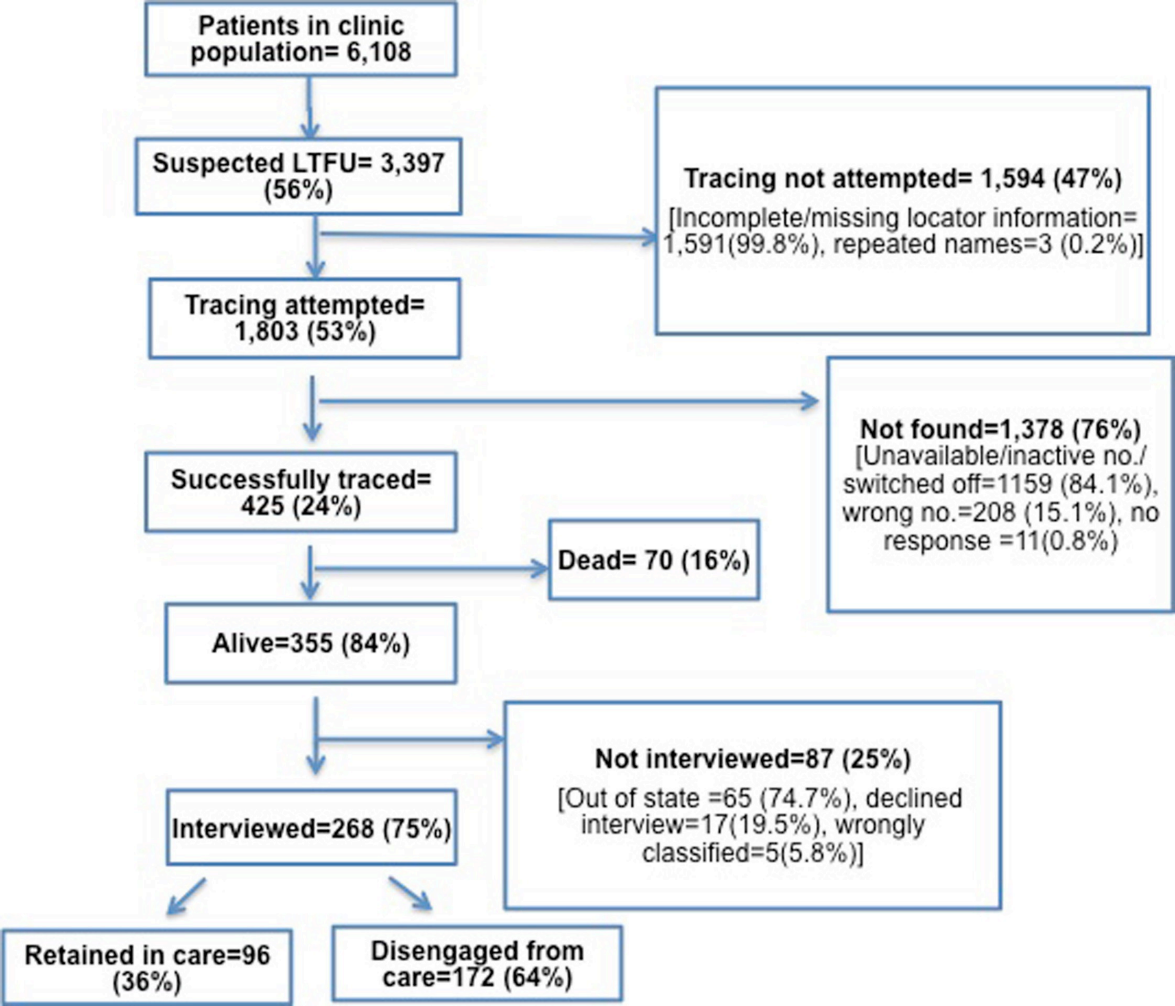
Flow chart depicting LTFU patients from LUTH HIV treatment program who enrolled into care from January 2010-December 2014.

A total of 6,108 patients enrolled into the program between 2010 and 2014, out of which 3,397 (56%) were LTFU. Tracing was attempted among 1,803 patients who had enough information for tracing and, of those, 425 (24%) were successfully traced. Two hundred and sixty-eight of the successfully traced patients consented to interviews. In total, 172 (64%) of the traced and interviewed patients reported being disengaged from HIV care while the remaining 96 (36%) reported having transferred to another treatment site to receive HIV care services (Fig 1).

### Characteristics of patients enrolled at LUTH

Table 1 presents baseline characteristics of 6,108 patients that received care between 2010 and 2014. The median age was 41 years (IQR: 35–47). They were mostly female (67%), married (58%), had secondary education or higher (71%) and median baseline CD4+ cell count of 229 cells/mm^3^ (IQR: 97–402).

In this larger patient cohort overall, lower proportions of patients LTFU were female, married, had tertiary-level education; a higher proportion were in WHO stage 4 of disease and they had lower median CD4+ cell count and higher median viral load compared to patients that were not LTFU.

**Table 1:** Baseline demographic and clinical characteristics of 6,108 patients enrolled into care in LUTH HIV treatment program from January 2010-December 2014.

Variables	All patients N = 6108	Patients not LTFU N = 2711	Patients LTFU N = 3397	p-value	Patients LTFU and not traced N = 2972	Patients LTFU and traced N = 425	p-value	Patients traced and not interviewed N = 157	Patients traced and interviewed N = 268	p-value
Median age, in years (IQR)	41(35–47)^a^	40(35–47)	41(35–47)	0.458	41(35–47)	40(34–47)	0.053	41(35–49)	39(34–45)	**0.037**
Female, n (%)	4065 (66.6)	1881(69.4)	2184 (64.3)	**<0.001**	1915(64.4)	269(63.3)	0.646	103(65.6)	168(62.7)	0.546
Marital status, n (%)										
Married Single Other* Missing	3032(58.2) 1457(28.0) 723(13.9) 896 (14.7)	1546(61.2) 648 (25.7) 331 (13.1)	1486 (55.3) 809 (30.1) 392 (14.6)	**<0.001**	1267 (54.3) 720 (30.6) 356 (15.1)	210(62.7) 89 (26.6) 36 (10.8)	**0.010**	69(58.5) 36(30.5) 13(11.0)	141(65.0) 53(24.4) 23(10.6)	0.451
Education, n (%)										
None Primary Secondary Tertiary Missing	507(9.7) 1004 (19.2) 2318 (44.4) 1397 (26.7) 882 (14.4)	211 (8.5) 419 (16.8) 1147 (45.9) 720 (28.8)	296 (10.9) 585 (21.4) 1171 (42.9) 677 (24.8)	**<0.001**	264(11.1) 529(22.2) 1017(42.7) 573(24.1)	32(9.3) 56(16.2) 154(44.5) 104(30.1)	**0.014**	16(12.6) 22(17.3) 51(56.5) 38(29.9)	16(7.3) 34(15.5) 103(47.0) 66(30.1)	0.327
WHO Stage, n (%)										
1 2 3 4 Missing	1210 (34.9) 685 (19.8) 1150 (33.2) 423 (12.2) 2640 (43.2)	747 (36.5) 415 (20.3) 716 (35.0) 169 (8.3)	462 (32.5) 270 (19.0) 434 (30.6) 245 (17.9)	**<0.001**	382(31.3) 234(19.2) 367(30.0) 239(19.6)	80(40.4) 36(18.2) 67(33.8) 15(7.6)	**<0.001**	21(28.0) 8(10.7) 36(48.0) 10(13.3)	59(48.0) 28(22.8) 31(25.2) 5(4.1)	**<0.001**
Median CD4, cells/mm^3^ (IQR)	229 (97–402)^b^	242 (123–392)	211 (78–411)	**<0.001**	199(73–398)	292(130–501)	**<0.001**	201(68–434)	334(199–529)	**<0.001**
Median viral load, copies/mL (IQR)	52951(3911–287143)^c^	47200(2686–236606)	57487(4934–337358)	**<0.001**	59856(4962–354400)	45231(4805–191000)	0.112	119593(16522–454038)	27800(2397–114032)	**<0.001**

*Divorced/Separated/Widowed

^a^Missing: 809 (13.2%)

^b^Missing: 522 (8.6%)

^c^Missing: 1650 (27%)

Among the cohort of LTFU patients, higher proportions of patients that were successfully traced were married, had tertiary education, were in WHO stage 1 and had higher median CD4+ cell count than those that could not be traced. The patients that were successfully traced and interviewed were slightly younger, had higher proportions of patients in WHO stage 1 and had higher median CD4+ cell count and lower median viral load compared to those that were not interviewed.

### Predictors of LTFU

Table 2 provides the adjusted and unadjusted HRs for the effect of patient characteristics on LTFU from the ART program. A total of 3,603 (59%) patients were included in the final model. Compared with married patients, single (never married) patients were 1.2 times more likely to become LTFU (aHR: 1.21, 95% CI: 1.08–1.36) and divorced, separated or widowed patients were 1.3 times more likely to become LTFU (aHR: 1.30, 95% CI: 1.12–1.50).

**Table 2:** Unadjusted and adjusted hazard ratios of being lost to follow-up among 6,108 patients enrolled into care in LUTH HIV treatment program from January 2010-December 2014.

Variables	Unadjusted HR (95% CI)	p-value	Adjusted HR^a^ (95% CI)	p-value	Adjusted HR^b^ (95% CI) (N = 3603)	p-value
Age (per 10 years)	0.99(0.94–1.03)	0.599	0.96(0.90–1.02)	0.177	1.00(0.95–1.05)	0.969
Sex						
Female	Ref		Ref			
Male	1.12(1.02–1.23)	0.020	1.06(0.94–1.19)	0.343	-	-
Marital status						
Married	Ref		Ref		Ref	
Single	1.23(1.10–1.37)	<0.001	1.17(1.03–1.33)	0.014	1.21(1.08–1.36)	**0.001**
Divorced/Separated/Widowed	1.21(1.05–1.40)	0.007	1.18(1.00–1.38)	0.046	1.30(1.12–1.50)	**<0.001**
Education						
None	Ref		Ref			
Primary	1.07(0.89–1.29)	0.481	1.20(0.97–1.47)	0.088	-	-
Secondary	0.86(0.72–1.02)	0.083	0.94(0.78–1.14)	0.523		
Tertiary	0.81(0.68–0.97)	0.024	0.89(0.72–1.08)	0.238		
Disease progression at baseline						
CD4≥200 and/or WHO Stage 1–2	Ref		Ref			
CD4<200 and/or WHO Stage 3–4	1.29(1.17–1.43)	<0.001	1.30(1.16–1.45)	<0.001	-	-
Year of enrollment						
2010	Ref		Ref			
2011	1.09(0.96–1.23)	0.192	3.15(2.74–3.61)	<0.001	-	-
2012	1.11(0.97–1.27)	0.122	1.04(0.89–1.20)	0.625		
2013	1.24(1.07–1.43)	0.004	0.88(0.71–1.08)	0.222		
2014	1.26(1.06–1.51)	0.009	1.21(0.99–1.48)	0.061		

HR: Hazards Ratio Ref: Reference category

^a^Full cox’s proportional hazard model

^b^Final cox’s proportional hazard model (Schoenfeld Residuals Test p = 0.062)

### Characteristics of interviewed cohort

Of the 268 traced and interviewed patients, 201 (75%) had initiated ART prior to discontinuation at LUTH and the remaining 67 (25%) had only been enrolled in HIV care services. Amongst the 201 patients that had previously initiated ART, 119 (44%) were still on ARV and reported sourcing ARVs from other clinics (77%) and friends (12%). Of the 119 that were still taking ART, 91 (77%) reported still being engaged in HIV care services at another clinic and 28 (23%) reported being disengaged from care. Of these 119 patients, 19 (16%) reported gaps in their treatment. The majority (92%) of the respondents that were still on ARVs had a 100% adherence over the prior 7 days and 66% said they never skip taking their ARVs (Table 3).

**Table 3:** Use of antiretroviral medication among 268 traced and interviewed LTFU patients.

Variables	N (%)
**Started on ARV**	
Yes	201 (75.0)
No	67 (25.0)
**Still on ART (n = 201)**	
Yes always	100 (49.8)
Yes sometimes	19 (9.5)
No	82 (40.8)
**Source of ARVs (n = 119)**	
Other clinic	91 (76.5)
Family	4 (3.4)
Friend	14 (11.8)
Pharmacy	10 (8.4)
**Doses of ARVs missed in last 7 days**^#^ **(n = 119)**	
None	110 (92.4)
1–4	5 (4.2)
All	4 (3.4)
**Last time ARV was missed (n = 119)**	
Within the past week	9 (7.6)
1–4 weeks ago	3 (2.5)
1–3 months ago	8 (6.7)
More than 3 months ago	20 (16.8)
Never skip ARVs	79 (66.4)

^#^ARVs mentioned by all respondents were once daily prescriptions

### Reasons for discontinuation in interviewed cohort

Of the 268 patients interviewed, the primary reported reason for discontinuation from LUTH was long distance to clinic (56%). In total, the most common reasons per category were: long distance to clinic (access to care, 88%), staff was not nice (clinic quality, 10%), busy at work (work and family, 3%), feeling healthy (medical, 14%) and not being permitted by religion or faith (alternate treatment and advice, 6%) [Table 4].

**Table 4:** Reasons for discontinuation of care from LUTH HIV treatment program among 268 traced and interviewed LTFU patients.

Category	Reasons mentioned	Ranked 1^st^ N (%) N = 268	Ranked 2^nd^ N (%) N = 217	Ranked 3rd N (%) N = 129	Total N (%) N = 268
**Access to care**	Long distance to clinic	150 (56.0)	65 (30.0)	22 (17.1)	237 (88.4)
	Long waiting time	12(4.5)	43 (19.8)	26 (20.2)	81 (30.2)
	Started treatment in another clinic	9 (3.4)	18 (8.3)	15 (11.6)	42 (15.7)
	High cost of transportation	11(4.1)	15 (6.9)	14 (10.9)	40 (14.9)
	High cost of test	5 (1.9)		3 (2.3)	8 (3.0)
	Others^a^	3 (1.1)	2 (0.9)	1 (0.8)	5 (1.9)
**Clinic quality**	Staff was not nice	5 (1.9)	8 (3.7)	13 (10.1)	26 (9.7)
	Afraid of scolding from clinic staff	2 (0.8)	5 (2.3)	6 (4.7)	13 (4.9)
	Attending clinic risked disclosure to community	2 (0.8)	5 (2.3)	4 (3.1)	12 (4.5)
	Others^b^	3 (1.1)	5 (2.3)	2 (1.6)	10 (3.7)
**Work and family**	Busy at work	9 (3.4)	17 (7.8)	10 (7.8)	36 (13.4)
	Busy caring for family	1 (0.4)	4 (1.8)	3 (2.3)	8 (3.0)
	Spouse/baby died	4 (1.5)	1 (0.5)		5 (1.9)
**Medical**	Feeling healthy	18(6.7)	12 (5.5)	7 (5.4)	37 (13.8)
	Didn’t need ARV	11(4.1)	4 (1.8)	2 (1.6)	17 (6.3)
	Others^c^	14 (5.2)	4 (1.8)	0 (0.0)	18 (6.7)
**Alternate treatment and advice**	Not permitted by religion or faith	9 (3.4)	7 (3.2)	0 (0.0)	16 (6.0)
	Others^d^	1 (0.4)	2 (0.9)	1 (0.8)	4 (1.5)

^a^ No work/no money, next scheduled clinic visit was far

^b^ Poor clinic environment, too many appointments, lack of privacy

^c^ Was sick, had side effects with ARV, does not have HIV, tired of ARV, did not understand importance, did not want caesarian section

^d^ Family person does not approve of clinic, started alternative medicine

### Reasons for stopping ART in interviewed cohort

Of the 82 patients that reported having stopped taking ART, the most common reasons given for stopping were long distance to clinic (32%), feeling healthy (30%), having finished ARVs without restocking (23%), not being permitted by faith/religion (16%), high transportation cost to clinic (15%) and not wanting to take ARVs for life (13%). Of the 19 patients that reported gaps in their treatment, the most common reasons given were long distance to clinic (47%), finishing ARVs without restocking (37%), high transportation cost to clinic (32%) and work responsibilities (26%) [Table 5].

**Table 5:** Reasons for discontinuation of ART for patients that discontinued from the LUTH HIV treatment program.

Variables	N (%)
Reasons for stopping ARVs* (n = 82)	
Finished ARVs without restocking	19 (23.2)
Suspected side effects of ARVs	6 (7.3)
Very weak/sick	4 (4.9)
Now on alternate medicine	5 (6.1)
Feeling healthy	25 (30.5)
High transportation cost to clinic	12 (14.6)
Long distance to clinic	30 (36.6)
Work responsibilities	8 (9.8)
Not ready to take ARVs for life	11 (13.4)
My religion/faith does not permit me	13 (15.9)
Others^a^	10 (8.4)
Reasons for treatment gaps* (n = 19)	
Finished ARVs without restocking	7 (36.8)
Forget to take ARVs	4 (21.1)
High transportation cost to clinic	6 (31.6)
Long distance to clinic	9 (47.4)
Work responsibilities	5 (26.3)
Others^b^	5 (26.3)

*Multiple responses allowed

^a^ Non-disclosure of HIV status, family person does not approve taking ARVs, high cost of tests, no longer pregnant, lack information, too much stress

^b^ Suspected side effects of ARVs, very weak/sick, family person does not approve taking ARVs, no work/no money

### Predictors of disengagement and re-engagement with care

Among the interviewed patients, bivariate analyses comparing characteristics of patients disengaged from HIV care to those still retained in HIV care outside LUTH revealed that significantly higher proportions of patients still not receiving care following LTFU from LUTH were male (73%, p = 0.020) and had not started ART in LUTH (85%, p<0.001). Additionally, a significantly higher proportions of patients that; are male (73%, p = 0.001), had not started ART in LUTH (79%, p<0.001), and had stayed longer duration in care before LTFU (82%, p = 0.017), were willing to re-engage in the LUTH antiretroviral program (Table A in S2 Appendix).

In multivariate analyses, the predictor of disengagement from care was not having started ART. The predictors of willingness to re-engage in care at LUTH were not having started ART, male sex and longer duration in HIV care prior to LTFU (Table 6).

**Table 6:** Multivariate analysis of characteristics associated with disengagement from care and willingness to re-engage into LUTH HIV treatment program among 268 traced and interviewed LTFU patients.

	Disengaged from care^a^ ^c^ (N = 268)				Willingness to re-engage^b^ ^d^ (N = 268)			
Characteristics	COR (95% CI)	p-value	AOR (95% CI)	p-value	COR (95% CI)	p-value	AOR (95% CI)	p-value
Age (per 10 years)	0.30(0.07–1.27)	0.103	0.11(0.09–1.33)	0.083	0.28(0.07–1.12)	0.073	0.43(0.70–2.61)	0.357
Sex								
Female	Ref		Ref		Ref		Ref	
Male	1.88(1.10–3.23)	0.021	1.49(0.80–2.79)	0.208	2.40(1.40–4.10)	0.001	1.91(1.05–3.45)	**0.033**
Marital status								
Married	Ref		Ref		Ref		Ref	
Single	1.72(0.82–3.63)	0.153	2.29(0.99–5.28)	0.052	0.80(0.41–1.56)	0.510	1.05(0.48–2.26)	0.907
Divorced/Separated/Widowed	0.85(0.40–1.78)	0.658	0.80(0.37–1.71)	0.559	1.02(0.48–2.16)	0.955	1.23(0.58–2.62)	0.594
Education								
None	Ref		Ref		Ref		Ref	
Primary	0.27(0.03–2.52)	0.253	0.25(0.03–2.26)	0.217	0.83(0.14–4.93)	0.841	0.57(0.08–4.26)	0.583
Secondary	0.38(0.04–3.23)	0.373	0.42(0.05–3.47)	0.419	0.66(0.12–3.53)	0.625	0.58(0.09–3.94)	0.581
Tertiary	0.22(0.23–1.88)	0.166	0.25(0.03–2.10)	0.203	0.48(0.09–2.61)	0.396	0.45(0.07–3.06)	0.417
ART initiation								
Started on ART	Ref				Ref		Ref	
Not started on ART	4.26(2.06–8.83)	<0.001	4.42(2.13–9.15)	**<0.001**	3.20(1.67–6.13)	<0.001	3.56(1.77–7.16)	**<0.001**
Months in care before LTFU (per 3 months)	1.00(1.00–1.00	0.929	1.00(1.00–1.00)	0.810	2.00(1.33–2.99)	0.001	2.14(1.36–3.36)	**0.001**

COR: crude odds ratio; AOR: adjusted odds ratio; Ref: Reference categories

Reference categories:

^a^Retained in care

^b^Not willing to re-engage in program

Box-Tidwell tests before transformation—age vs. disengagement from care (p = 0.494, linear relationship); months in care vs. disengagement from care (p = 0.026, non-linear relationship); age vs. willingness to re-engage (p = 0.360, linear relationship); months in care vs. willingness to re-engage (p = 0.832, linear relationship)

Hosmer-Lemeshow goodness-of-fit test:

^c^p = 0.625;

^d^p = 0.326

In subsequent follow-up, 95 of the 268 patients interviewed returned to LUTH for care services.

## Discussion

In this study conducted at a high-volume HIV clinic in southern Nigeria, we found a high proportion of LTFU. Of the patients that were traced and interviewed, majority were truly disengaged from care and not receiving ART from any source. We discovered that the most common reason for discontinuation from HIV care from the large clinic, stopping ART, or having treatment gaps (for those still on ART) was long distance to the clinic.

The LTFU rate found in this evaluation was consistent with that reported in other studies of LTFU for patients receiving HIV care services in sub-Saharan Africa (2.6–57.4%) [20]. Our rate was similar to that reported from programs in north central Nigeria with rates of 56.4% and 58% in hub and spoke sites respectively [13], but higher than a study from the southeast with rates of 32.8% and 11% in public and private programs, respectively [3].

A possible explanation for the regional differences is that the private southeastern program takes efforts to trace lost patients [3], along with the increased mobility of people in metropolitan areas of the southwest region [21]. Our value was higher than the LTFU rate of 28% from an earlier study involving part of this patient cohort, as that earlier study focused only on patients on ART [12]. Being unmarried was a predictor of being LTFU in our study, similar to findings from other studies [12,22], implying that having a committed spousal support system could be a motivation for patients to overcome existing barriers to continuing care.

The rate of true disengagement from care and ART among interviewed patients in this study was higher than in similar studies in sub-Saharan Africa [6,8,17,19]. This is particularly worrisome as patients not receiving care are at risk of progressive disease and death when they do not have access to life-saving medication and continual care, possibly evidenced by the outcome of death among 16% of the traced patients in our study; a rate of mortality lower than pooled estimates of 39% in sub-Saharan Africa [23]. In addition, those that were previously on ART are at an increased risk of developing and transmitting drug resistant strains of HIV [3].

Similar to other studies, we found patients in our study cohort reported that long distance to clinic was a major reason for discontinuation of HIV care and stopping ART [19,24]. Prior studies have shown that patients are more likely to remain in HIV care when ART services are decentralized to centers closer to patients’ homes [25–27]. The cohort evaluated in this study originally enrolled at LUTH when ART services were not decentralized. Several patients also reported having discontinued care and stopping ART because they were feeling healthy. This could occur among patients when they increase in weight, have high CD4+ cell count or undetectable viral load [28,29]; the dangers of discontinuing ART even when feeling healthy should be stressed during health education sessions.

An encouraging finding for the LUTH clinic was that only few of the interviewed patients discontinued care secondary to clinic quality such as poor clinic environment and lack of privacy. This is interesting because the clinic environment was compromised by a fire incident in 2012 without being adequately rehabilitated [30] and may be due to the subjective sense of connection patients have to each other, the clinic’s site, providers and processes [31].

Similar to other studies, we found that patients that were not yet on ART were more likely than those on ART to be disengaged from care [32]. Our finding underscores the importance of the test and treat strategy to aid retention in care, which was adopted in Nigeria in 2016 [14]. Further research could examine any difference in LTFU and disengagement rates in the current test and treat strategy era as well as the re-engagement to care kinetics within the ART program.

Most of the patients interviewed were willing to return to the LUTH clinic and 95 of them were linked back to care. This positive attitude despite identified barriers to continued care is encouraging and could potentially translate to high re-engagement in care once barriers are addressed. It is not immediately clear why female patients were less willing to return to care and this would require exploratory research. Those patients with long duration in HIV care prior to LTFU might be challenged by long duration of physical symptoms, psychosocial distress, co-morbidities, social stigma, treatment fatigue, as well as the adverse effects that may be associated with ART [33–35], which could result in their unwillingness to return to care.

Some limitations must be considered in our study. First, our findings cannot be generalized to all the LTFU patients within the cohort because a significant number of them could not be traced as a result of missing and inaccurate locator information in the secondary data or as a result of having moved out of state resulting in a potential bias. A salient recommendation would be updating and verification of patient information at each follow-up visit. Our results thus pertain to only those patients that were traced and interviewed as they differed in some regard to those that were not interviewed. Secondly, our definition of retention in care subsequent to LTFU from LUTH was based on self-report of attendance in other facilities other than LUTH. This may be considered simplistic as it does not account for frequency or regularity of visits or use of ART [36].

In conclusion, retention in HIV care is a major challenge in HIV/AIDS management programs in sub-Saharan Africa. There might be site-based variability in reasons for discontinuation and outcomes of patients LTFU and our understanding of these in the Nigerian setting is limited. Our study fills in this gap in knowledge by describing the status as well as willingness to re-engage in care of patients fitting the criteria of LTFU from one the largest ART programs in the country. It is crucial that ART programs incorporate LTFU tracing routinely into their care [9,10,37] and further research should explore innovative ways of re-engaging patients back into care in our setting. In addition to early tracing, well-organized community support initiatives are also effective strategies [38–40]. In settings where services have been decentralized, the flexibility in the system should be properly communicated to patients so they understand their options for receiving treatment and care. Health information systems should also be strengthened and patient information linked across the antiretroviral programs (including the decentralized sites) in the nation in order to enhance the documentation of transfers and to further facilitate continuity in care. The findings of our study have implications for the strengthening of healthcare systems to address the needs of a large population of HIV-infected patients.

## Supporting information

S1 Appendix Original survey questionnaire. (DOCX)

S2 Appendix Bivariate analyses of characteristics associated with disengagement from care and willingness to re-engage in program. (DOCX)

S3 Appendix Dataset for original clinic population of 6,108. (XLSX)

S4 Appendix Dataset for 268 traced and interviewed LTFU patients. (XLSX)
