# Supplementary material for: Status of HIV-infected patients classified as lost to follow up from a large antiretroviral program in southwest Nigeria
Source: PLoS One. 2019 Jul 25;14(7):e0219903. doi: 10.1371/journal.pone.0219903 (PMC6657856; doi:10.1371/journal.pone.0219903)
Supplement: S1 Appendix — (DOCX) [file pone.0219903.s001.docx]

**QUESTIONNAIRE**

Patient ID:………………….

Interviewer’s name:…………………………………… Questionnaire no:………

Time started:…… Time ended:……..

**Interviewer:** Circle the letter in front of the selected answer(s).

Do not read responses unless the directions indicate.

**Statement of Person Giving Consent (Respondent)**

I have read or heard the description of the research and I have had it translated into a language I understand. I have also talked it over with the researcher to my satisfaction and I understand that my participation is voluntary. I know enough about the purpose, methods and benefits of the research study to judge that I want to take part in it. I understand that I may freely stop being part of this study at any time.

**DATE: _________________ SIGNATURE: _____________________**

**PERSONAL CHARACTERISTICS**

1. How old are you as at last birthday? _____ years
2. Sex
3. Male
4. Female
5. Marital status

a) Single

b) Married

c) Separated

d) Divorced

e) Widowed

1. Religion

a) Christianity

b) Islam

c) Traditional

d) Others (specify) _________________________

1. Ethnicity

a) Yoruba

b) Igbo

c) Hausa

d) Others (specify) _________________________

1. Level of education

a) No formal education

b) Primary school uncompleted

c) Primary school completed

d) Secondary school uncompleted

e) Secondary school completed

f) Post secondary (specify)_____________

g) Religious schooling only

h) Literacy classes only

1. Employment status
2. Employed (Go to question 8)
3. Unemployed (Go to question 9)
4. If employed, what is your occupation?

a) Senior professional e.g. Doctor, Lawyer, Accountant etc

b) Intermediate professional e.g. Nurses, Teachers, Secretaries etc

c) Junior professional/skilled e.g. Tailor, Typist, Auxiliary Nurses, Hairdressers,

Caterers etc

d) Semi skilled e.g. Traders, Ward maids, School assistants, Food vendors, Nannies etc

e) Unskilled e.g. Cleaners, House helps, Labourers, Farmers etc

1. If unemployed, are you?
2. Housewife
3. Students
4. Apprentices
5. Retiree
6. Other:

**OUTCOME STATUS OF PATIENTS LTFU**

1. When last were you at APIN clinic__________(month/year)
2. Why did you stop coming to the clinic? **(Multiple responses allowed. Rank the top 3)**
3. High costs of transportation __
4. Clinic is too far __
5. The waiting time is too long __
6. I travelled __
7. I moved __
8. I started treatment in another clinic __
9. Afraid of scolding from clinic staff __
10. Attending clinic risked disclosure to community __
11. Staff was not nice __
12. Too many appointments __
13. Lack of privacy __
14. I am busy at work __
15. I am busy caring for family __
16. Attending clinic risked disclosure to family __
17. Family person does not approve of clinic __
18. I didn’t need ARVs __
19. I am feeling healthy __
20. My religion/faith does not permit me __
21. Other:
22. Were you ever placed on treatment with antiretroviral medicine in the clinic?
    1. Yes (Go to question 13)
    2. No (Go to question 16)
23. If yes, are you still using the medicine?
    1. Yes always (Go to question 14)
    2. Yes sometimes (Go to question 14)
    3. No (Go to question 15)
24. If yes, where do you get your medicine from? **(Multiple responses allowed. Rank the top 3)**
    1. My other clinic __
    2. Pharmacy outside of clinic __
    3. My spouse __
    4. My friend __
    5. Other (specify):
25. If No to Q13, why are you not using the medicine? **(Multiple responses allowed. Rank the top 3)**
    1. Suspected side effects of ARVs __
    2. Very weak/sick __
    3. Now on alternate medicine______________(specify) __
    4. I am feeling healthy __
    5. High transportation cost to clinic __
    6. Travelled away __
    7. Work responsibilities __
    8. Not ready to take ARVs for life __
    9. Lack information about ARVs __
    10. Non-disclosure of HIV status __
    11. Family person does not approve taking ARVs __
    12. My religion/faith does not permit me __
    13. Other:
26. If Yes sometimes to Q13, why are you not always using the medicine? **(Multiple responses allowed. Rank the top 3)**
    1. Suspected side effects of ARVs __
    2. Very weak/sick __
    3. I forget to take ARVs __
    4. I am feeling healthy __
    5. High transportation cost to clinic __
    6. Travelled away __
    7. Work responsibilities __
    8. Not ready to take ARVs for life __
    9. Lack information about ARVs __
    10. Non-disclosure of HIV status __
    11. Family person does not approve taking ARVs __
    12. My religion/faith does not permit me __
    13. Other:
27. In the past 7 days, how many doses of your ARVs have you missed?

| Name of ARV Drug | # Pills each time (Pills per dose) | # Times per day  (Doses per day) | # of Doses missed |
| --- | --- | --- | --- |
|  |  |  |  |
|  |  |  |  |
|  |  |  |  |

1. When was the last time you missed any of your ARVs?
   1. Within the past week
   2. 1-2 weeks ago
   3. 2-4 weeks ago
   4. 1-3 months ago
   5. More than 3 moths ago
   6. Never skip ARVs
2. Are you registered for HIV care in another clinic?
   1. Yes : specify clinic_____________
   2. No
3. Would you be willing to return for care in APIN clinic?
   1. Yes
   2. No
4. If no, why not? **(Multiple responses allowed. Rank the top 3)**
   1. High costs of transportation __
   2. Clinic is too far __
   3. The waiting time is too long __
   4. I travelled __
   5. I moved __
   6. I started treatment in another clinic __
   7. Afraid of scolding from clinic staff __
   8. Attending clinic risks disclosure to community __
   9. Staff was not nice __
   10. Too many appointments __
   11. Lack of privacy __
   12. I am busy at work __
   13. I am busy caring for family __
   14. Attending clinic risks disclosure to family __
   15. Family person does not approve of clinic __
   16. I don’t need ARVs __
   17. I am feeling healthy __
   18. My religion/faith does not permit me __
   19. Other:

Thank you for your time.

___________________________________________________________________

**CLINICAL & LABORATORY PARAMETERS**

**BASELINE(Date…...) MOST RECENT(Date……..)**

Viral load: Viral load:

CD4 count: CD4 count:

Urea: Urea:

Creatinine: Creatinine:

Alanine transaminase Alanine transaminase

Weight: Weight:

BMI: BMI:

WHO Stage: WHO Stage:
