# Supplementary material for: Status of HIV-infected patients classified as lost to follow up from a large antiretroviral program in southwest Nigeria
Source: PLoS One. 2019 Jul 25;14(7):e0219903. doi: 10.1371/journal.pone.0219903 (PMC6657856; doi:10.1371/journal.pone.0219903)
Supplement: S2 Appendix — (DOCX) [file pone.0219903.s002.docx]

Table A: Bivariate analyses of characteristics associated with disengagement from care and willingness to re-engage in program among 268 traced and interviewed LTFU patients

|  | **Disengagement from care** | | | **Willingness to re-engage** | | |
| --- | --- | --- | --- | --- | --- | --- |
| **Variables** | **Yes (%)**  **N=172** | **No (%)**  **N=96** | **p-value** | **Yes (%)**  **N=162** | **No (%)**  **N=106** | **p-value** |
| **Age group (years)**  <35  35-44  ≥45  **Median age (IQR)** | 41(56.9)  82(66.1)  49(68.1) | 31(43.1)  42(33.9)  23(31.9) | 0.314  0.233 | 39(54.2)  76(61.3)  47(65.3) | 33(45.8)  48(38.7)  25(34.7) | 0.381  0.140 |
| **Sex**  Female  Male | 99(58.9)  73(73.0) | 69(41.1)  27(27.0) | **0.020** | 89(53.0)  73(73.0) | 79(47.0)  27(27.0) | **0.001** |
| **Marital status**  Married  Single  Divorced/Separated/Widowed | 120(62.8)  32(74.4)  20(58.8) | 71(37.2)  11(25.6)  14(41.2) | 0.281 | 117(61.3)  24(55.8)  21(61.8) | 74(38.7)  19(44.2)  13(38.2) | 0.793 |
| **Education**  None  Primary  Secondary  Tertiary | 6(85.7)  23(62.2)  88(69.3)  55(56.7) | 1(14.3)  14(37.8)  39(30.7)  42(43.3) | 0.152 | 5(71.4)  25(67.6)  79(62.2)  53(54.6) | 2(28.6)  12(32.4)  48(37.8)  44(45.4) | 0.445 |
| **Started on ARV**  Yes  No | 115(57.2)  57(85.1) | 86(42.8)  10(14.9) | **<0.001** | 109(54.2)  53(79.1) | 92(45.8)  14(20.9) | **<0.001** |
| **Months in care prior to LTFU**  0 – 12  13 – 24  25 – 36  37 – 48  >48  **Median number of months (IQR)** | 90(59.6)  26(72.2)  22(68.8)  20(62.5)  14(82.4)  9.5(0-29) | 61(40.4)  10(27.8)  10(31.2)  12(37.5)  3(17.7)  4(0-25.5) | 0.271  0.090 | 80(53.0)  21(58.3)  22(68.8)  25(78.1)  14(82.4)  13(0-34) | 71(47.0)  15(41.7)  10(31.3)  7(21.9)  3(17.6)  2.5(0-20) | **0.017**  **0.001** |
